# VERU-111 Promotes an Anti-Tumor Response Through Restoration of Gut Microbial Homeostasis and Associated Metabolic Dysregulation

**DOI:** 10.3390/cells15020141

**Published:** 2026-01-13

**Authors:** Md Abdullah Al Mamun, Ahmed Rakib, Mousumi Mandal, Wei Li, Duane D. Miller, Hao Chen, Mitzi Nagarkatti, Prakash Nagarkatti, Udai P. Singh

**Affiliations:** 1 Department of Pharmaceutical Sciences, College of Pharmacy, The University of Tennessee Health Science Center, 881 Madison Avenue, Memphis, TN 38163, USAmmandal1@uthsc.edu (M.M.);; 2 Department of Pathology, Microbiology & Immunology, School of Medicine, University of South Carolina, 6311 G Ferry Road, Columbia, SC 29209, USA

**Keywords:** colorectal cancer, gut microbiota, dysbiosis, metabolic pathways, restoration, anti-cancer effect

## Abstract

The rising global burden of colorectal cancer (CRC) has now positioned it as the third most common cancer worldwide. Chemotherapy regimens are known to disrupt the composition of the gut microbiota and lead to long-term health consequences for cancer patients. However, the alteration of gut microbiota by specific chemotherapeutic agents has been insufficiently explored until now. The purpose of this study was to assess changes in the gut microbiota following treatment with VERU-111 as a chemotherapy agent for the treatment of CRC. We thus performed a metagenomic study using 16S rRNA gene amplicon sequencing of fecal samples from different experimental groups in the azoxymethane (AOM) and dextran sodium sulfate (DSS)-induced murine model of CRC. To predict the functional potential of microbial communities, we used the resulting 16S rRNA gene sequencing data to perform Kyoto Encyclopedia of Genes and Genomes (KEGG) pathway analysis. We found that the administration of VERU-111 led to a restructured microbial community that was characterized by increased alpha and beta diversity. Compared to the mice treated with DSS alone, VERU-111 treatment significantly increased the relative abundance of several bacterial species, including *Verrucomicrobiota* species, *Muribaculum intestinale*, *Alistipes finegoldii*, *Turicibacter*, and the well-known gut-protective bacterial species *Akkermansia muciniphila*. The relative abundance of *Ruminococcus*, which is negatively correlated with immune checkpoint blockade therapy, was diminished following VERU-111 administration. Overall, this metagenomic study suggests that the microbial shift after administration of VERU-111 is associated with suppression of several metabolic and cancer-related pathways that might, at least in part, facilitate the suppression of CRC. These favorable shifts in gut microbiota suggest a novel therapeutic dimension of using VERU-111 to treat CRC and emphasize the need for further mechanistic exploration.

## 1. Introduction

The mammalian intestinal tract is closely packed with its own microbial ecosystem that accommodates over 1000 operational taxonomic units (OTUs) and more than 40 trillion microorganisms [1,2]. This microbiome serves as a vital regulator that shapes and educates key elements of the host’s innate and adaptive immune responses, which in turn regulate and sustain vital aspects of the symbiosis between host and microbes [3]. The microbiome is also deeply involved in regulating other key physiological functions, including vitamin production, altering immune response, pathogen resistance, and nutrient absorption. Consequently, disruptions in the composition and diversity of the microbiome (known as dysbiosis) are associated with a range of diseases, including cancers [4]. There is also mounting evidence that tumors themselves harbor bacteria and maintain a complex tumor ecosystem [5]. Dysbiosis of gut microbiota (GM) can be measured using different established and well-reported indices like alpha and beta diversity. For instance, alpha diversity of the fecal microbiome represents an intra-sample measure of the abundance and phylogenetic relatedness of microbial communities in the gut, with higher alpha diversity often correlating with improved health outcomes in specific disease conditions [6,7]. On the other hand, beta diversity measures the diversity among the samples and is a useful tool to answer the question of how different the microbial communities are in a study.

The intestinal microbiome plays a crucial role in both the development of cancer and therapeutic responses to cancer [8]. Mechanistic studies reveal that GM can influence the development of colorectal cancer (CRC) by interacting with colonic epithelial cells and host immune response via the production of various metabolites, proteins, and macromolecules [9]. Disruption of this microbial ecosystem can cause a significant threat to the health of the host, leading to the development of not only CRC but also gastrointestinal disorders and neurological, respiratory, metabolic, hepatic, and cardiovascular conditions [10]. Extensive evidence suggests that the GM of CRC patients is distinct from that of healthy individuals and is marked by an increase in harmful taxa and a reduction in protective ones [11]. This altered GM is not only involved in intestinal inflammation and tumor development but also in manipulating the anti-cancer immune response [12].

Disruption of the microbiome compromises the responsiveness of subcutaneous tumors, including EL4 lymphoma, MC38 colon carcinoma, and B16 melanoma models to immunotherapy and platinum-based chemotherapy, while an intact commensal microbiome is required for an optimal response by myeloid-derived cells [13]. Many gut microbial populations, including *Gammaproteobacteria* species, contribute to the development of chemoresistance to gemcitabine therapy in solid tumors [14]. *Fusobacterium nucleatum*, previously recognized for its significant role in tumor initiation and progression, also impacts the risk of CRC, dysplasia, and the outcomes of cancer treatment. Lower levels of *F. nucleatum* are associated with improved prognosis of CRC and higher survival of CRC patients [15,16].

The GM strongly influences the effectiveness of immune checkpoint inhibitors (ICIs) and treatment-associated side effects [17,18,19]. ICI therapy is a pivotal approach for the treatment of CRC, particularly the CRC subtype associated with deficient mismatch repair, high microsatellite instability (dMMR/MSI-H), and metastatic potential [20,21]. The distinct biological potential of bacteria for improving immunotherapy responses has been explored recently. A synthetic probiotic engineered from *Pediococcus pentosaceus* showed efficacy against CRC with a significant reduction in tumor burden [22]. Also interlinked with CRC growth are GM-derived metabolites, including bile acids, tryptophan metabolites, and short-chain fatty acids, which also modulate the immune response [23]. These effects of GM-derived metabolites are context-dependent, and their immunoregulatory functions extend beyond the intestinal compartment to act systemically via both cellular and metabolic pathways [24]. In this study, we used KEGG pathway analysis to obtain a clear picture of changes in metabolic processes after VERU-111 treatment in DSS-induced CRC.

A significant knowledge gap remains in understanding the extent to which chemotherapeutic agents influence the homeostasis of the gut microbiome and alterations in microbiome, in turn, affect the efficacy, toxicity, and overall outcomes of CRC therapies. Addressing this uncertainty and gap is crucial for optimizing treatment strategies and developing novel drug therapies. Our drug candidate, VERU-111, is a selective colchicine binding site inhibitor that shows strong antitumor efficacy in multiple solid tumors, including melanoma, breast cancer, lung cancer, pancreatic cancer, and prostate cancer [25,26,27,28,29]. Our previous study (data under review) revealed the potential of VERU-111 as a treatment for CRC to elicit better outcomes and suppress tumor burden by upregulation of apoptosis, activation of T cells, and downregulation of the programmed cell death protein 1 (PD-1)/programmed cell death ligand 1 (PD-L1) axis. This study was designed to provide a comparative analysis of the alterations in GM composition following exposure to a carcinogenic agent and subsequent treatment with VERU-111. By examining these microbial shifts, we sought to understand how changes in the GM may influence the therapeutic efficacy of VERU-111 in the context of CRC. Our findings highlight the critical role of GM dynamics in modulating treatment outcomes and underscore the importance of considering microbial factors in the development of effective therapies for CRC and other cancers.

## 2. Materials and Methods

### 2.1. Animal Studies

Female 6–8-week-old C57BL/6 mice were procured from Jackson Laboratories (Bar Harbor, ME, USA). On arrival at our facility, all mice were acclimated for one week under standard housing conditions, including a 12 h light/dark cycle, in a pathogen-free animal facility at our institution. All experiments were performed under a protocol (number 23–0450) approved by our institution’s Institutional Animal Care and Use Committee (IACUC). After the 1-week acclimatization period, mice were randomly divided into control, DSS, and VERU-111-treated experimental groups, each of which contained five mice (*n* = 5/group). Mice in the control group received normal drinking water throughout the experimental period and received an injection of vehicle alone (PBS) on day 1, while the mice in the DSS and VERU-111 groups received a subcutaneous injection of 10 mg/kg of Azoxymethane (AOM) and were provided with 2.5% dextran sulfate sodium (DSS) in drinking water from day 3 to day 10, followed by a 10-day recovery period with normal drinking water. This DSS/water cycle was repeated 3 times. Starting from the 6th week, the mice in the treatment group were administered 10 mg/kg VERU-111 via oral gavage 5 days per week for 3 weeks, while mice in the control and DSS groups were administered the vehicle (30% PEG300 in water) orally throughout the treatment period at the same dosing frequency as the VERU-111 group. Body weight and clinical indicators of colitis-associated CRC, including diarrhea, stool consistency, and the presence of blood in feces, were monitored daily until the experimental endpoint.

### 2.2. Fecal Sample Collection and DNA Isolation

At the endpoint of the experimental protocol at day 60, fecal samples were collected immediately from all mice in 2 mL Eppendorf tubes. All animals were kept in a separate cage to avoid intragroup cross-contamination during the collection of samples. The samples were placed on ice after collection and then stored at −80 °C immediately until further analysis. Frozen aliquots (200 mg) of each fecal sample were thawed, and genomic DNA was extracted using a Qiagen QIAamp DNA Stool Mini Kit (Cat. no. 51604; Qiagen, Valencia, CA, USA) in accordance with the manufacturer’s protocol. Immediately upon isolation, the concentration and quality of DNA were determined using a Nanodrop spectrophotometer (Thermo Scientific, Waltham, MA, USA). Extracted DNA was preserved at −80 °C until further analysis, when it was sent for 16S rRNA sequencing to our collaborator at another institution.

### 2.3. 16S rRNA Amplicon Sequencing

Total gut microbiota profiling was performed with a microbiome analysis platform using QIIME 2 (2025.7)/VSEARCH 16S paired end pipeline. A detailed procedure for 16S rRNA microbial profiling has been described previously [30]. Bacterial DNA libraries of the 16S rRNA V3-V4 hypervariable regions were made by amplification with added Illumina adapter overhang nucleotide sequences prior to sequencing on the Illumina MiSeq platform. The Nephele platform (https://nephele.niaid.nih.gov) from the National Institute of Allergy and Infectious Diseases (NIAID) Office of Cyber Infrastructure and Computational Biology (OCICB; Bethesda, MD, USA) was used to analyze the sequenced reads [31]. Output files were further analyzed with the LefSe Galaxy web application tool developed by the Huttenhower group (Harvard University, Cambridge, MA, USA) to evaluate total gut microbial composition [32]. The PICRUSt2 software package was used to perform KEGG pathway analysis of our 16S rRNA gene amplicon sequencing data at levels 1, 2, and 3 to produce heatmaps that show the predicted changes in GM function for control, DSS, and VERU-111-treated animals.

### 2.4. Statistical Analysis

All data are presented as mean values ± standard error of the mean (SEM). Statistical analyses were performed using either one-way analysis of variance (ANOVA) followed by Tukey’s multiple comparison tests (as indicated in the figure legends) or Student’s *t*-test, depending on the experimental groups, to determine the significance level. A *p*-value of 0.05 was the level of significance used in all analyses (* *p* < 0.05, ** *p* < 0.01, *** *p* < 0.001, and **** *p* < 0.0001). Graphs were generated using GraphPad Prism software; Version 9.0 (Boston, MA, USA).

## 3. Results

### 3.1. VERU-111 Treatment Reversed Overall Gut Microbiota Dysbiosis

**Figure 1:**
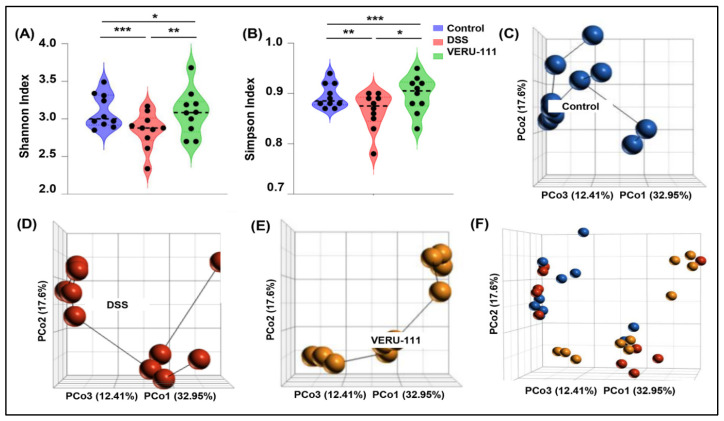
Overall representation of gut microbial diversity alteration in different experimental groups (*n* = 10). Alpha diversity was analyzed using (**A**) the Shannon index and (**B**) the Simpson index. Violin plots show distribution, with individual samples represented as dots. Principal coordinate analysis (PCoA) was conducted on bacterial beta diversity based on the Bray–Curtis dissimilarity of gut microbial populations and is represented as three-dimensional PCoA plots of the (**C**) Control, (**D**) DSS, and (**E**) VERU-111 groups. (**F**) Combined PCoA plot including overlaid PCoA representation of all three groups. Percent variance explained by PCo1, PCo2, and PCo3 is indicated on the corresponding axes. Statistical significance was determined using one-way ANOVA with Tukey’s multiple comparison tests (* *p* < 0.05, ** *p* < 0.01, and *** *p* < 0.001).

Given that the alteration in the composition and function of microbiota contributes to the induction of CRC, we assessed changes in alpha and beta diversity of the gut microbiome under different experimental conditions. We evaluated the modulation that results after treatment of experimental mice with the novel anti-cancer agent VERU-111 and observed that VERU-111 treatment significantly improved gut microbial alpha diversity, as evidenced by improved Shannon and Simpson indices when compared to DSS alone (Figure 1A,B). Next, we analyzed beta diversity across the three experimental groups and performed principal coordinate analysis (PCoA), which revealed a clear separation among the GM of the different experimental groups. Each group was situated in a separate area of the PCoA axis, indicating a change in the overall structure of the GM on induction of CRC with AOM/DSS and treatment with VERU-111. The Bray–Curtis coefficient (PCo) calculates the dissimilarity between the samples based on the abundance of different taxa in each group of samples. We observed a significant difference in the GM composition in the different experimental groups using the Bray–Curtis model (Figure 1C–F). Taken together, our data suggest that VERU-111 improves both α- and β-diversity of the GM, underscoring its safety and potential to enhance chemotherapeutic efficacy in the context of CRC.

### 3.2. VERU-111 Modulated Gut Microbiota and Favored Beneficial Bacterial Phyla

**Figure 2:**
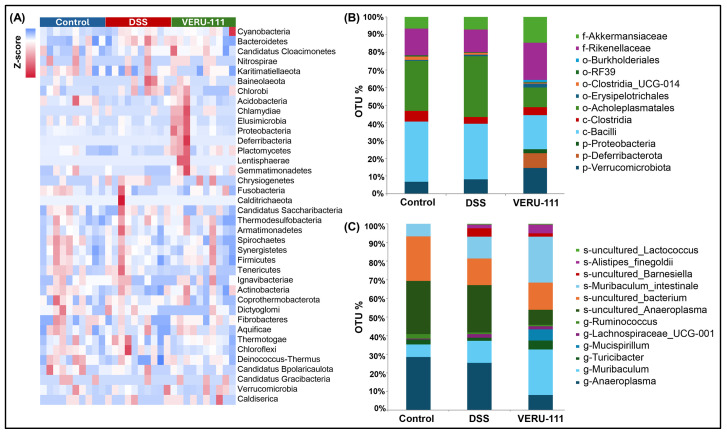
Relative abundance of bacteria at different taxonomical levels in different experimental groups. (**A**) Heatmap of gut microbiota abundance across three experimental groups. Shown are Z-score-normalized relative abundances of bacterial taxa (*n* = 10). (**B**) Phylum-to-family level community distribution. Stacked bar plots depict relative abundances at higher taxonomic levels. (**C**) Genus and species-level composition analysis. Stacked bar plots depict the relative abundance of dominant genres across groups.

Previous reports describe a notable difference in overall gut microbial composition at the phylum level between healthy individuals and individuals with CRC [33]. Samples from healthy individuals were enriched in *Planctomycetes* species, while samples from individuals with CRC contained elevated levels of *Bacteroides* and *Dorea* species [34]. The potent anti-cancer and cytotoxic metabolite-producing taxon *Actinobacteria* plays a pivotal role in influencing tumor suppression [35]. In the current study, a gut microbiota abundance heatmap analysis of samples from mice treated with VERU-111 revealed phylum-level changes in the GM, including a marked increase in beneficial bacterial populations of *Actinobacteria* and *Planctomycetes* after treatment and a noticeable reduction in harmful and CRC-associated taxa *Bacteroidetes* and *Baineolaeota* (Figure 2A). Next, we analyzed microbial alterations across taxonomic levels, from phylum to species; the most significantly increased or dysregulated populations are shown (Figure 2B,C). Interestingly, treatment with VERU-111 produced a clear restorative effect on DSS-induced dysbiosis, as shown by consistent shifts in microbial composition across both taxonomic analyses. Towards this, we observed a marked increase in *Verrucomicrobiota* that corresponded to a rise in *Akkermansiaceae* at the family level in the VERU-111-treated group compared with the group treated with DSS alone (Figure 2B). At the genus and species levels, we observed an increased abundance of *Muribaculum intestinale* bacteria, which are known to be beneficial for CRC outcomes, and a significant reduction in CRC-associated detrimental taxa *Anaeroplasma* (Figure 2C). Collectively, these findings suggest that VERU-111 not only mitigates DSS-induced dysbiosis but also fosters a microbiome that is enriched in protective, health-promoting bacterial communities, underscoring its therapeutic potential in shaping gut microbial ecology.

### 3.3. VERU-111 Enhanced the Abundance of Protective Bacteria and Created a Tumor-Suppressing Gut Environment

**Figure 3:**
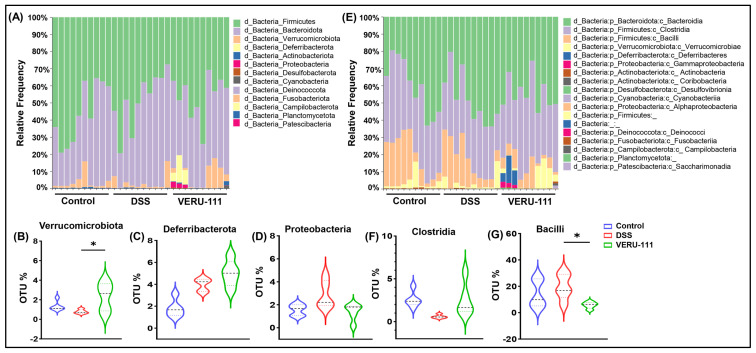
Phylum-level changes in gut microbiota in three experimental conditions. (**A**) Stacked bar plots of phylum-level microbial community structure depict the relative abundance of major bacterial phyla across the Control, DSS, and VERU-111 groups. DSS exposure shifted the gut microbial composition towards dysbiosis, characterized by a decreased abundance of the Firmicutes and *Verrucomicrobiota* phyla and an expansion of dysbiosis-associated phyla, including *Proteobacteria* and *Deferribacterota*. VERU-111 treatment partially restored microbial balance, increasing beneficial phyla and reducing DSS-induced expansion. (**B**–**D**) Quantitative comparison of selected phyla. (**B**) Violin plots show a significant increase in *Verrucomicrobiota* after VERU-111 treatment. (**C**) *Deferribacterota* levels trended higher in mice treated with DSS and VERU-111, but the difference was not statistically significant. (**D**) *Proteobacteria* were elevated in mice treated with DSS but reduced after VERU-111 treatment. (**E**) Class-level microbial distribution. Stacked bar plots illustrate the relative abundance of major bacterial classes in three different experimental groups. (**F**) DSS challenge had no notable effect, while VERU-111 slightly increased the level of *Clostridia*. (**G**) The violin plot represents a significant reduction in the frequency of *Bacilli* upon VERU-111 treatment. Violin plots represent mean ± SEM. Statistical significance was determined using one-way ANOVA followed by Student’s *t*-test (* *p* < 0.05).

**Figure 4:**
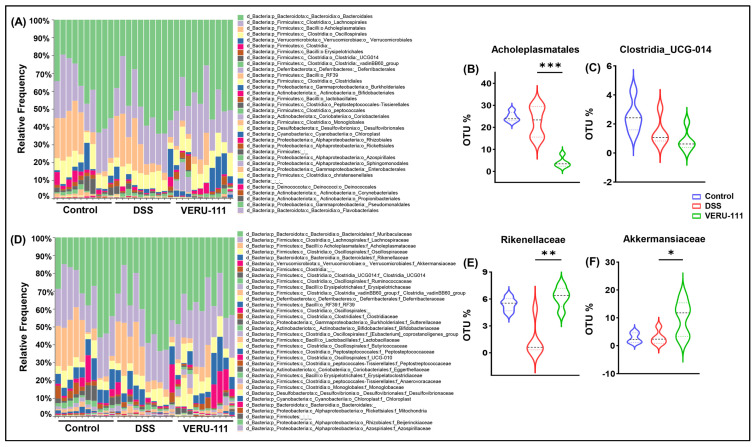
Impact of VERU-111 treatment on gut microbiota composition and alteration of specific bacterial taxa. (**A**) Stacked bar plots showing the relative abundance of bacterial taxa across three experimental groups at the order level. (**B**) Violin plot showing the percentage of Operational Taxonomic Units (OTUs) for *Acholeplasmatales*. (**C**) *Clostridia_UCG-014* OTU % showed no significant differences among groups. (**D**) Stacked bar plots show the relative abundance of bacterial taxa across three experimental groups at the family level. In the VERU-111-treated mice, violin plots show (**E**) a significant increase in *Rikenellaceae* and (**F**) significantly elevated OTU% for *Akkermansia*. Violin plots represent mean values ± SEM. Statistical analysis was performed using one-way ANOVA followed by Student’s *t*-test. (* *p* < 0.05, ** *p* < 0.01, *** *p* < 0.001).

These promising results led us to conduct a detailed analysis of the relative frequency of each taxon after VERU-111 treatment. The data revealed an increased relative abundance of *Verrucomicrobiota*, a modest reduction in *Proteobacteria*, and no appreciable change in *Deferribacterota* (Figure 3A–D). At the class level, we observed no significant change in class *Clostridia*, whereas we observed a notable suppression of Gram-positive class *Bacilli* in the VERU-111-treated group (Figure 3E–G). Given that the functional role of microbes within the class *Bacilli* is multifaceted, with some species reported to confer protective effects and others implicated in CRC progression and adverse outcomes [36,37], we evaluated the changes in the GM at the order and family levels, aiming to make a comprehensive summary of the effects of VERU-111 treatment. We observed a pronounced reduction in bacteria from the order *Acholeplasmatales* (Figure 4A,B). To date, there is limited evidence supporting either a positive or a negative role for bacteria from this order in modulating CRC or influencing therapeutic interventions, including immunotherapy. Given the sharp decrease in this population and our findings of tumor suppression after VERU-111 treatment, the shift in the order *Acholeplasmatales* may provide new insights into its potential role in CRC. Consistent with this observation, we observed at the family level a decrease in the abundance of family *Acholeplasmataceae* (order *Acholeplasmatales*) (Figure 4D). Overall, we also observed an increase in many healthy GM flora, including members of the *Rikenellaceae* and *Akkermansiaceae*, following treatment with VERU-111 (Figure 4E,F). Members of the family *Akkermansiaceae* are well known for their protective role, and *Akkermansia muciniphila* has been reported as a promising immunomodulatory microbiome in the context of CRC. However, the role of bacteria from the *Rikenellaceae* family is still controversial [38]. Taken all together, VERU-111 showed a gut environment enriched in protective bacteria that might serve to suppress tumors, at least in part. However, further detailed investigation will be required to draw a prudent conclusion.

### 3.4. VERU-111 Improved the Abundance of Gut-Friendly Bacteria to Increase the Effectiveness of Chemotherapy

**Figure 5:**
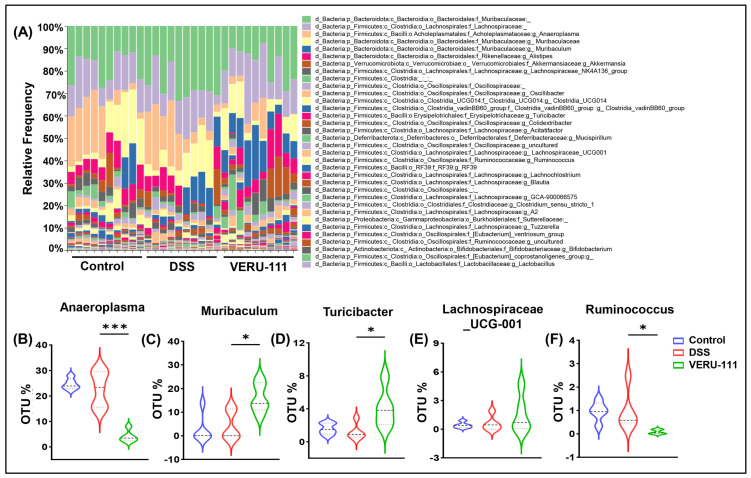
VERU-111 restored the abundance of commensal gut genera that were altered by administration of DSS. (**A**) Taxonomic composition analysis showing the relative abundance of the major bacterial genera in fecal samples from three experimental groups. (**B**–**F**) The relative abundance of key genera was significantly altered by VERU-111 treatment. Violin plots depict the distribution of OTU percentage for (**B**) *Anaeroplasma*, (**C**) *Muribaculum*, (**D**) *Turicibacter*, (**E**) *Lachnospiraceae UCG-001*, and (**F**) *Ruminococcus*. Violin plots represent mean values ± SEM. Statistical analysis was performed using one-way ANOVA followed by Student’s *t*-test. (* *p* < 0.05, *** *p* < 0.001).

We then assessed genus and species richness to identify specific bacteria that are enriched or depleted on VERU-111 treatment. In congruence with our family-level data, we observed a remarkable increase in genus *Akkermansia* and a decrease in genus *Anaeroplasma* on treatment of mice with VERU-111 (Figure 5A,B). We also observed a significant increase in genus *Muribaculum* in the VERU-111-treated group relative to that treated with DSS alone (Figure 5C). A notable increase in genus *Muribaculum* is positively correlated with CRC, as *Muribaculum* species have the potential to sustain intestinal homeostasis via utilization of mucin-derived monosaccharides [39]. The VERU-111-treated group also exhibited an increase in *Turicibacter* and a decrease in *Ruminococcus* but no notable changes in *Lachnospiraceae* relative to the mice treated with DSS alone (Figure 5C–F). *Turicibacter* species exert protective effects in the host by stimulating the production of beneficial microbial metabolites, including short-chain fatty acids such as butyrate, and in vitro promote ROS-mediated apoptosis [40].

**Figure 6:**
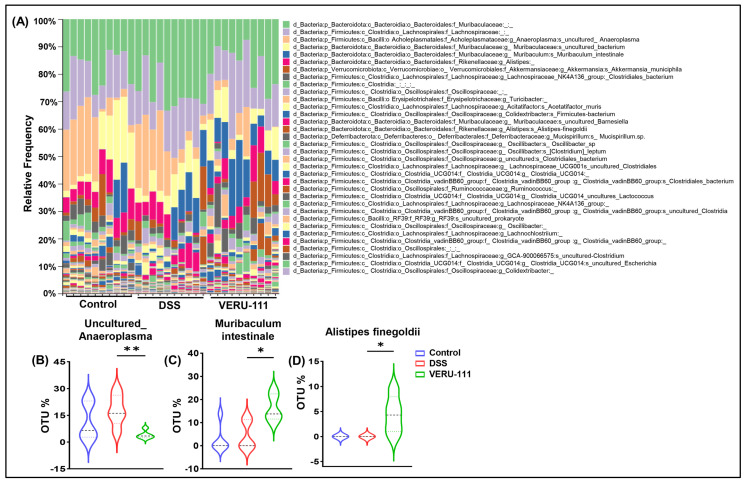
Treatment with VERU-111 restores specific healthy gut microbial species altered by DSS. (**A**) Taxonomic composition analysis showing the relative abundance of the most prevalent bacterial species in fecal samples from three groups of experimental mice. Violin plots depict the distribution of OTU percentage for (**B**) *Uncultured Anaeroplasma*, (**C**) *Muribaculum intestinale*, and (**D**) *Alistipes finegoldii*. Violin plots represent mean ± SEM. Statistical analysis was performed using one-way ANOVA followed by Student’s *t*-test. (* *p* < 0.05, ** *p* < 0.01).

We observed a significant decrease in the abundance of uncultured *Anaeroplasma* species in the VERU-111-treated group relative to that in the DSS group (Figure 6A,B). *Anaeroplasma* is associated with increased inflammation and is present at elevated abundance in samples from patients with untreated CRC [41]. *Muribaculum intestinale* is a healthy GM species that is well recognized for its positive correlation with producing butyrate and reducing cancer cachexia [42]. The abundance of *Alistipes finegoldii* is associated with an improved response to immunotherapy and boosts antitumor immunity in solid tumors [43]. In this study, we observed a substantial increase in the populations of *Muribaculum intestinale* and *Alistipes finegoldii* at the species level (Figure 6C,D). Collectively, these findings suggest that VERU-111-induced alterations in the GM profile are beneficial modulations that boost the pharmacological efficacy of VERU-111 as a chemotherapeutic and provide a foundation for the development of novel microbiota-targeted therapeutic strategies.

### 3.5. Prediction of Functional Capacity of Microbiome Through KEGG Pathway Analysis

**Figure 7:**
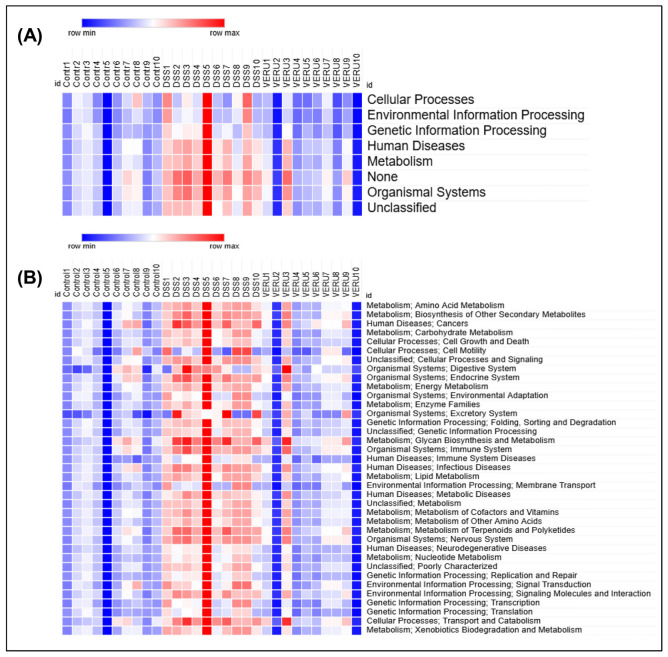
Predicted key functional profiles of gut microbiota altered by VERU-111 treatment. Heatmap analysis of KEGG functional pathways predicted from 16S rRNA gene sequencing data. (**A**) Heatmap shows clustering of broad functional categories (Level 1) across Control, DSS, and VERU-111-treated groups. (**B**) Detailed heatmap shows specific metabolic and cellular pathways (Level 2). The color scale represents the relative abundance of each functional category in each sample, ranging from blue (row minimum) to red (row maximum). Overall, the DSS group exhibited a distinct functional profile compared to the Control group, while VERU-111 treatment shifted the profile toward the Control phenotype.

We used Kyoto Encyclopedia of Genes and Genomes (KEGG) pathway analysis to generate heatmaps that illustrate the potential functional capacity of GM communities that were altered after VERU-111 treatment. In the DSS-induced CRC, GM functional pathways were markedly dysregulated, with elevated activity in disease-related pathways, cellular metabolic processes, and environmental information processing pathways. Treatment with VERU-111 substantially normalized these functional alterations, particularly in metabolism and genetic information processing pathways (Figure 7A). Further analysis within each major shift revealed that metabolic function, including amino acid, lipid, carbohydrate, terpenoid, and polyketide metabolism, was upregulated in the mice with DSS-induced CRC. In contrast, treatment of these mice with VERU-111 normalized that pattern toward that observed in naïve mice. Moreover, DSS-challenged mice exhibited a pronounced increment of pathways related to membrane transporters and human disease-related modules, which were reversed through VERU-111 treatment (Figure 7B).

**Figure 8:**
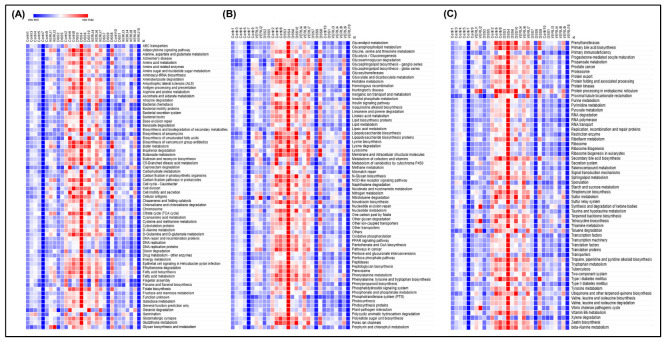
Detailed heatmap of KEGG Level 3 pathways showed a homeostatic restoration of key metabolic pathways in mice treated with VERU-111. (**A**–**C**) KEGG Level 3 functional pathways across all samples (Control, DSS, and VERU-111 groups). Color intensity represents the relative abundance of each pathway (Red = high abundance, Blue = low abundance). This visualization delves into in-depth information on the functional shifts induced by DSS and their subsequent restoration by VERU-111 treatment.

At level 3 KEGG pathway analysis for specific metabolic processes, we observed significant upregulation of several metabolic pathways, including biotin, glycolipid, and lipoic acid metabolism, biosynthesis of folate and the aromatic amino acids phenylalanine, tyrosine, and tryptophan, and degradation of xylene in DSS-challenged mice relative to naïve mice. Notably, in mice treated with VERU-111, these pathways were restored to normal homeostatic levels (Figure 8A–C). Together, these findings suggest that VERU-111 elicits a corrective effect on microbiome functional dysregulation that contributes to the restoration of gut microbial homeostasis.

## 4. Discussion

Rich microbial diversity and high population levels are hallmarks of a healthy GM, but this diversity becomes highly dysregulated when a mammalian host experiences one of many pathological conditions, including CRC [44,45]. Moreover, most of the currently available chemotherapeutics induce dysbiosis in GM, which ultimately facilitates other adverse reactions [46,47]. In this study, we used the experimental animal model of CRC to show that mice treated with VERU-111 demonstrated a marked healthy shift that reversed the dysbiosis caused by DSS. Findings from this study confirm that, unlike conventional chemotherapeutics used in CRC management, VERU-111 maintains a healthy GM population. We showed that mice receiving DSS + VERU-111 resulted in an increased microbial diversity and enrichment of beneficial taxa, alongside a reduction in potentially disease-friendly taxa. Such a shift in microbial diversity is consistent with an improved immune-responsive gut microbial ecosystem and improved responsiveness to chemotherapy or ICI therapy like anti-PD-1. VERU-111 treatment normalized several DSS-induced metabolic pathway dysregulations, including those affecting lipid metabolism and folate biosynthesis. A major protective mechanism exerted by a healthy GM is the production of short-chain fatty acids, including butyrate, which is associated with disease-free survival [48]. Higher bacterial diversity, including commensal bacterial richness, is strongly associated with higher disease-free survival with CRC [49]. In this study, DSS-challenged mice exhibited an induction of dysbiosis and fecal samples with lower bacterial abundance, while treatment with VERU-111 reversed the dysbiosis and improved both alpha and beta diversity. *Akkermansia muciniphila* bacteria have a symbiotic effect that accelerates the production of butyrate by butyrogenic bacterial taxa [50]. Interestingly, we found a notable increase in the *Akkermansiaceae* and the *Rikenellaceae* families on treatment with VERU-111. The class *Clostridia* species *Blautia wexlerae* exerts a similar symbiotic effect that assists other GM species to produce propionate and butyrate, which are excreted in the feces [51]. Relative to mice treated with DSS alone, mice treated with VERU-111 exhibited an increased abundance of *Clostridia* and of *Muribaculum intestinale*. In a mouse model of cancer cachexia, *Muribaculum intestinale* is significantly depleted in the GM, leading to a disease condition characterized by diminished butyrate production that improved on supplementation with *Muribaculum intestinale* [42]. Taken together, these data indicate that VERU-111 treatment restructured the gut microbiome by increasing bacterial diversity and promoting the expansion of butyrate-producing taxa, changes that are likely to contribute to its tumor-suppressive effects.

During cancer, GM profiles can serve as predictive biomarkers of treatment efficacy and prognosis [12]. The crosstalk between the GM and chemotherapy could significantly advance our understanding of cancer therapy and patient outcomes, but this area of research is still limited. Chemotherapy induces GM dysbiosis and perturbs the integrity of the gastrointestinal mucosal barrier, thereby accelerating mucosal inflammation and resulting in chemotherapy-induced gastrointestinal mucositis [52]. A clinical study shows that GM alterations in 94 patients who received a standard 5-Fluorouracil-based adjuvant chemotherapy following radical surgery for advanced CRC reported an increased abundance of *Fusobacterium nucleatum*, which is related to negative outcomes in CRC [53]. Bacteria from the phylum *Proteobacteria* can metabolize the chemotherapeutic drug gemcitabine into its inactive form (2′,2′-difluorodeoxyuridine) [14]. In the present study, we did not detect any major changes in the *Fusobacteria* or *Proteobacteria* phyla. The family *Akkermansiaceae* species *Akkermansia muciniphila* boosts the effectiveness of many chemotherapeutics like FOLFOX (oxaliplatin, fluorouracil, and calcium folinate) in CRC patients [54]. A combination of tyrosine kinase inhibitors and ICI therapy potentiates the immunotherapy response by increasing the growth of *Muribaculum* and its metabolite, urocanic acid, which ultimately reduces the recruitment of myeloid-derived suppressor cells (MDSCs) via the CXCL1-CXCR2 axis [55]. Our findings of a higher abundance of *Akkermansiaceae* and *Muribaculum* in fecal samples from mice in the VERU-111-treated group than those treated with DSS alone corroborate these earlier findings.

The efficacy of immunotherapy is strongly affected by the GM, which modulates host immune responses and participates in the remodeling of the tumor microenvironment [56]. In preclinical studies, mice harboring a favorable GM exhibit significantly enhanced responses to anti-PD-L1 therapy than those carrying an unfavorable GM. This advantage can be conferred exogenously via cohousing or fecal transplant [57]. The loss of GM diversity is associated with poor treatment outcomes of ICI therapy, whereas enrichment of bacterial flora, including *Faecalibacterium*, *Bifidobacterium*, *Lactobacillus*, and *Akkermansia muciniphila*, has a favorable impact on ICI therapy [57,58,59,60]. Further, another study reported a higher abundance of bacteria in the genus *Turicibacter* in a patient who was responsive to anti-PD-1 therapy, relative to that in the non-responsive patient cohort [61]. In our study, mice subjected to VERU-111 treatment exhibited a favorable GM diversity characterized by improved abundance of healthy bacteria, including members of the family *Akkermansiaceae* and the genus *Turicibacter* (phylum *Bacillota*, formerly known as *Firmicutes*). In a clinical trial, patients with anti-PD-1-refractory melanoma who received a fecal microbiota transplant from a patient who responded to anti-PD-1 therapy demonstrated a better response, increased activation of CD8^+^ T cells, and suppression of interleukin-8-expressing myeloid cells [62]. Together, these preclinical and clinical studies highlight the role of GM in shaping the response to ICI therapy, with distinct effects being observed with different bacterial taxa. Our study depicted the richness of ICI therapy-favorable taxa in mice treated with VERU-111 with DSS relative to those treated with DSS alone. Future mechanistic studies are required, which may incorporate a paradigm shift that targets the modulation of the gut microbiota to optimize immunotherapy response in CRC patients and perhaps all cancer patients.

The response of patients to cancer therapy varies substantially from person to person and is closely associated with host immune status [63,64,65]. Importantly, growing evidence confirms the influence of GM on tumor immunity [66]. Beneficial commensal microbes augment both innate and adaptive immunity, while cancer-associated dysbiosis can dampen the host immune response [67]. The GM plays a crucial role in shaping patients’ responses to cancer therapies via interactions with diverse immune cell populations [68]. For example, GM-derived metabolites and altered metabolic pathways play a crucial role in regulating immune cells, including T cells, B cells, dendritic cells, and macrophages [69]. Short-chain fatty acids produced by organisms in the GM augment CD8^+^ T cell effector functions by modifying their cellular metabolism [70]. In this study, KEGG pathway analysis of the 16S RNA sequencing data from samples in the different experimental groups depicted a dysregulation in metabolism and human disease-related pathways in response to DSS insult. In mice treated with DSS alone, metabolism of amino acids, lipids, carbohydrates, terpenoids, and polyketides was upregulated. This is not surprising since lipid metabolism is frequently upregulated in cancer to support high-energy demands and the rapid growth of tumor cells, allowing the tumor cells to proliferate and resist apoptosis [71,72]. Folic acid plays an important role in cellular regulation, and during CRC, the folate metabolic pathway is upregulated to facilitate the rapid growth of tumor cells [73]. Data from our KEGG pathway analysis of DSS-treated mice corroborates a similar pattern of upregulated biosynthesis of folate and aromatic amino acids, metabolism of biotin and lipoic acid, and degradation of xylene. Interestingly, treatment of mice with DSS and VERU-111 restored these pathways to more normal levels. Thus, by suppressing these metabolic pathways, VERU-111 may reinforce its intended therapeutic effect of lowering tumor burden. Taken together, our data indicate that VERU-111 treatment restores the DSS-induced dysregulation of metabolic pathways and perturbs key pathways that supply energy to tumor cells, thereby promoting their death and suppressing tumorigenesis. A limitation of this study is that, based on data at our disposal, we cannot draw a prudent conclusion regarding the precise mechanisms by which VERU-111 alters the gut microbiota. We acknowledge that additional mechanistic studies and clinical validation are required to draw a prudent conclusion regarding the mechanistic pathways.

## 5. Conclusions

In summary, in an experimental murine model of CRC, VERU-111 improved both alpha and beta diversity of the GM diversity, characterized by an increased proportion of healthy bacterial taxa. These alterations of the microbiome and the restoration of dysregulated metabolic pathways by VERU-111 are novel and depict the safety of this drug candidate. These data also indicate that VERU-111 may enhance the efficacy of conventional chemotherapy and immunotherapy for the treatment of CRC. Moreover, our study outcomes provide a rationale for the development of future therapeutic approaches that integrate microbiome-targeted intervention with standard CRC therapy, potentially improving treatment outcomes.

## Data Availability

The raw data described in this manuscript will be made available to the public by the authors, without any reservation.
